# Effects of Combined Music and Caffeine on Psychophysiological Responses During Exercise: A Systematic Review and Exploratory Meta-Analysis

**DOI:** 10.3390/nu18203441

**Published:** 2026-10-20

**Authors:** Qinshuo Zhang, Honghan Xu, Junli Mo, Fucong Wang, Xing Gao, Chengyu Zhou, Zhuo Zeng, Xusong Dong, Dongxu Huang, Haoqiang Shi, Haoran Li, Kai Gao, Yongmin Xie, Aiguo Zhou, Diandong Lang, Bingbing He

**Affiliations:** 1 School of Sports and Exercise, Beijing Normal University, Beijing 100088, China; 15210977888@163.com (Q.Z.);; 2 School of Leisure Sports and Tourism, Beijing Sport University, Beijing 100084, China; 3 School of Physical Education, Zhengzhou University, Zhengzhou 450000, Chinalangdiandong123@126.com (D.L.); 4 School of Wushu, Henan University, Kaifeng 475000, China; 5 School of Strength and Conditioning Training, Beijing Sport University, Beijing 100084, China2024210137@bsu.edu.cn (Z.Z.); 2023012202@bsu.edu.cn (X.D.); dongxuhuang1027@163.com (D.H.); m15966507473@163.com (H.S.); xieyongminbsu@163.com (Y.X.); aiguozhou@126.com (A.Z.); 6 College of Sports and Health, Linyi University, Linyi 276000, China; 7 Department of Sports Science, The University of Suwon, Hwaseong-si 18323, Republic of Korea; 8 School of Physical Education and Sports, Beijing Normal University, Beijing 100875, China

**Keywords:** music, caffeine, exercise, systematic review, meta-analysis

## Abstract

**Background**: This systematic review and exploratory meta-analysis evaluated the effects of combined music and caffeine on psychophysiological responses during exercise. **Methods**: PubMed, Embase, Web of Science, and the Cochrane Library were searched from database inception to 10 June 2026. Randomized crossover studies comparing caffeine plus music with placebo plus no music, caffeine alone, or music alone were eligible. Outcomes included rating of perceived exertion (RPE), Physical Activity Enjoyment Scale (PACES), Feeling Scale (FS), and heart rate (HR). Findings were synthesized descriptively by outcome and comparator, considering study-specific effect estimates, confidence intervals, and experimental conditions. Random-effects meta-analyses were retained as secondary exploratory summaries. Because fewer than 10 independent studies were available, meta-regression and formal assessment of small-study effects were not undertaken. **Results**: Five crossover studies involving 88 participants were included. Study-specific RPE estimates varied from large reductions to estimates close to the null or in the opposite direction. PACES point estimates favoured the combined intervention, but their magnitudes differed substantially in comparisons with placebo plus no music and caffeine alone. Feeling Scale and heart-rate findings also varied across studies. All four primary meta-analyses showed I^2^ values exceeding 90%, and the certainty of evidence was very low for all primary outcomes. **Conclusions**: The available evidence comprises five small crossover studies conducted in different populations and experimental settings. Study-specific findings suggest possible improvements in exercise enjoyment, while perceived exertion, affective valence, and heart-rate responses vary across studies. Neither the descriptive findings nor the secondary exploratory pooled estimates establish a stable, broadly applicable magnitude of intervention benefit. Given the very low certainty of evidence, no firm recommendations can be made regarding the use of caffeine combined with music across exercise populations and settings. Further adequately powered studies using clearly specified interventions and comparable outcome assessments are needed to determine which responses are reproducible and practically meaningful.

## 1. Introduction

During exercise, the decision to continue, slow down, or stop is not determined only by muscle function or cardiorespiratory capacity [1]. How hard the exercise feels, whether the activity is enjoyable, and whether the individual feels mentally activated also play important roles in shaping exercise behavior [2]. For this reason, psychophysiological outcomes such as rating of perceived exertion (RPE), exercise enjoyment, Feeling Scale (FS), and heart rate (HR) are increasingly used to evaluate the practical value of exercise-related interventions [3,4]. These outcomes are especially meaningful because they capture both the subjective experience of exercise and the physiological response that accompanies physical effort [5].

Caffeine has long been used as a simple and accessible ergogenic aid. Its effect is generally linked to stimulation of the central nervous system, increased alertness, and reduced perception of fatigue [6]. In exercise settings, caffeine may allow individuals to tolerate a given workload with less discomfort or maintain effort for a longer period. However, its influence is not limited to performance outcomes [7]. Changes in perceived exertion, arousal, and cardiovascular response may partly explain why caffeine improves exercise capacity in some situations but shows weaker or inconsistent effects in others [8]. Factors such as dose, timing, habitual caffeine use, training background, and exercise mode may further shape these responses.

Music is another widely used strategy during exercise, but it works through a different pathway. Rather than directly acting as a nutritional stimulant, music can alter attention, emotion, rhythm, and motivation [9]. A well-matched piece of music may reduce the focus on bodily discomfort, make exercise feel more pleasant, and help individuals maintain movement rhythm. These effects make music particularly relevant to RPE, PACES, and FS. In some contexts, music may also influence HR indirectly through emotional activation or changes in exercise intensity [10]. Therefore, music is not merely a background stimulus; it may actively reshape how exercise is perceived and experienced.

The combination of music and caffeine is theoretically plausible because the two strategies may act through complementary pathways. Caffeine can increase alertness, attenuate fatigue-related sensations, and improve several aspects of exercise performance, with the most consistent evidence supporting doses of 3–6 mg/kg body mass. However, the magnitude of the response varies considerably between individuals because of factors such as habitual caffeine intake, genetic variation, and differences in supplementation protocols [11]. Music may act through attentional, motivational, affective, and rhythmic pathways, potentially making exercise feel more pleasant or less effortful. Combining these strategies could therefore influence both the physiological and perceptual dimensions of exercise, although an additive or synergistic effect should not be assumed without direct evidence [12].

Participant sex and female-specific physiological factors may also contribute to variation in the response to caffeine. This issue is particularly relevant to the present review because three of the five included studies enrolled exclusively female samples. A recent systematic review by Fornaresio et al. evaluated ten randomized, double-blind, placebo-controlled trials in female athletes using caffeine doses of 3–6 mg/kg. Ergogenic effects were reported across different exercise modalities and menstrual-cycle phases, although relatively few studies directly compared multiple phases, and phase-specific adverse responses and substantial inter-individual variability remained possible [13]. Menstrual-cycle phase, hormonal-contraceptive use, habitual caffeine intake, and individual tolerance should therefore be considered when interpreting studies involving female participants. These factors were not reported consistently enough in the included studies to support reliable moderator analyses.

Nevertheless, the current evidence on the combined intervention remains limited. Existing trials differ in caffeine dosage, music tempo or preference, exercise protocol, participant characteristics, and comparator design. These differences make it difficult to determine whether caffeine combined with music provides an additional benefit beyond either component alone. Therefore, this systematic review and exploratory meta-analysis aimed to evaluate the effects of caffeine combined with music on RPE, PACES, FS, and HR during exercise.

## 2. Materials and Methods

### 2.1. Design and Reporting

The review was structured according to the PRISMA reporting framework [14]. The review was registered in the International Prospective Register of Systematic Reviews (PROSPERO; registration number CRD420261431625).

### 2.2. Literature Search

A systematic search was conducted in PubMed, Embase, Web of Science, and Cochrane Library. The search covered all records available from database inception to 10 June 2026. Reference lists of relevant articles and reviews were also checked to identify additional studies that may not have been captured through database searching. The search strategy combined four concept groups: caffeine, music, exercise, and psychophysiological outcomes. Search terms included caffeine-related terms, such as “caffeine” and “coffee”; music-related terms, such as “music,” “auditory stimulation,” and “rhythmic auditory stimulation”; exercise-related terms, such as “exercise,” “physical activity,” “sport,” and “training”. The complete electronic search strategies, search dates, database platforms, and numbers of records retrieved are presented in Supplementary Table S1.

### 2.3. Eligibility Criteria

Eligible participants were adolescents or adults who completed an exercise task under controlled experimental conditions. Healthy individuals, athletes, and recreationally active participants were eligible. Studies were required to use a randomized, counterbalanced, or controlled crossover design in which the same participants completed a caffeine-plus-music condition and at least one eligible comparator condition.

The intervention was the combined administration of caffeine and exposure to music before or during exercise. Caffeine could be provided as capsules, drinks, gum, or another clearly described caffeine-containing product. Music could be self-selected, preferred, or selected by the investigators.

The primary comparator was placebo plus no music. Secondary comparators were caffeine without music and music without caffeine. The eligible outcomes were rating of perceived exertion, Physical Activity Enjoyment Scale scores, Feeling Scale scores, and heart rate.

Reviews, observational studies, case reports, animal studies, non-exercise studies, and conference abstracts without sufficient numerical data were excluded. Studies were also excluded when caffeine or music was not clearly described or when the effect of the combined intervention could not be separated from other co-interventions.

When a study reported multiple exercise tests or assessment points for the same outcome, the measurement obtained after the principal exercise test was selected. If the study did not identify a principal test, the final post-exercise measurement was used. This selection rule was applied without reference to the direction or statistical significance of the result.

### 2.4. Data Extraction

Data were independently extracted by two reviewers using a standardized extraction form. Disagreements were resolved through discussion. Extracted information included first author, publication year, country, study design, intervention order, washout period, sample size, participant characteristics, exercise protocol, caffeine dose and timing, music characteristics, exact intervention and comparator conditions, outcome scale, and assessment point.

For each eligible comparison, we extracted the condition-specific means and standard deviations, the mean and standard deviation of the within-participant differences where available, and paired test statistics, confidence intervals, *p* values, or participant-level data that could be used to calculate the sampling variance of the paired effect estimate.

Each study contributed no more than one effect estimate to an individual meta-analysis. Multiple exercise tests, assessment points, or comparator conditions involving the same participants were not treated as independent observations. The three comparator contrasts were synthesized separately.

### 2.5. Risk-of-Bias Assessment

Risk of bias was independently assessed by two reviewers using the Cochrane risk-of-bias framework [15], with additional considerations specific to crossover trials. The assessment covered random-sequence generation, allocation concealment, blinding, incomplete outcome data, selective reporting, intervention order, washout periods, period effects, and potential carryover effects. Disagreements were resolved through discussion.

The certainty of evidence for each primary outcome was assessed using the GRADE framework [16]. Risk of bias, inconsistency, indirectness, imprecision, and risk of missing evidence were considered.

### 2.6. S Data Synthesis and Statistical Analysis

During revision, structured descriptive synthesis was made the principal basis for interpreting the findings. Studies were organized by outcome and the predefined comparator contrasts. The direction, magnitude, and 95% confidence interval of each study-specific estimate were considered alongside sample size, participant characteristics, caffeine regimen, music intervention, and exercise protocol. Findings were not summarized by counting statistically significant results, and differences between study contexts were not treated as established effect modifiers. The previously conducted random-effects analyses were retained as secondary exploratory summaries, presented alongside the constituent study estimates. These pooled values were not used as the principal basis for conclusions about intervention effectiveness or the benefit expected in a particular population or exercise setting. The independent study was the unit of analysis. All included studies used crossover designs; therefore, effect estimates and their sampling variances were calculated using within-participant paired data.

Three comparator-specific contrasts were defined before synthesis. The primary contrast was caffeine plus music versus placebo plus no music, whereas the secondary contrasts were caffeine plus music versus caffeine without music and caffeine plus music versus music without caffeine. For each outcome, a study contributed no more than one paired effect estimate to each comparator-specific meta-analysis. Because effect estimates involving different comparator conditions from the same crossover trial shared both participants and the caffeine-plus-music condition, these estimates were statistically correlated. We therefore conducted separate pairwise meta-analyses for the three comparator contrasts and did not include multiple correlated estimates from the same trial in a single model or calculate an overall pooled effect across comparators. No formal multiplicity adjustment was applied across the comparator-specific analyses because they addressed distinct, predefined questions. Accordingly, the reported 95% confidence intervals are pointwise rather than simultaneous, and findings from the secondary contrasts were considered exploratory and interpreted cautiously.

For each study, the effect estimate was calculated from the within-participant difference between the caffeine-plus-music and comparator conditions. Standardized mean differences were used because of differences in outcome scales or measurement procedures across studies. A small-sample correction was applied. Sampling variances were obtained from reported or reconstructed paired standard errors and therefore incorporated the within-participant correlation between conditions.

Where the standard deviation of the within-participant differences was available, the standard error of the mean difference was calculated as:

SE(MD) = SDdiff/√n,

where SDdiff was the standard deviation of the participant-level differences and n was the number of participants completing both conditions. When SDdiff was not directly reported, paired confidence intervals, paired test statistics, *p* values, or participant-level data were used to reconstruct the paired standard error. Where necessary, SDdiff was calculated as:

SDdiff = √(SDE^2^ + SDC^2^ − 2rSDE SDC),

where r represented the within-participant correlation between the experimental and comparator conditions.

Study-specific paired effect estimates and standard errors were pooled using generic inverse-variance random-effects models. Effect estimates are reported as standardized mean differences with 95% confidence intervals. Between-study heterogeneity was assessed using Cochran’s Q and the I^2^ statistic. Because I^2^ is imprecisely estimated when few studies are available, heterogeneity statistics were interpreted cautiously.

No subgroup analysis or meta-regression was undertaken because fewer than 10 independent studies were available. Funnel plots, Egger-type tests, trim-and-fill analyses, and other formal analyses of small-study effects were not conducted for the same reason. Publication bias therefore could not be statistically assessed and cannot be excluded. Leave-one-out analyses were not used as evidence of robustness. All analyses were performed using Stata version 15 (StataCorp LLC, College Station, TX, USA).

## 3. Results

### 3.1. Study Selection

A total of 1111 records were identified through database searches, including PubMed (*n* = 962), Embase (*n* = 94), the Cochrane Library (*n* = 37), and Web of Science (*n* = 18). After the removal of 107 duplicates, 1004 records remained for title and abstract screening. Of these, 994 records were excluded because they did not meet the eligibility criteria. Ten full-text articles were assessed, of which five were excluded. Five crossover studies [17,18,19,20,21] were included in the systematic review and meta-analysis (Figure 1).

### 3.2. Study Characteristics

Five crossover studies involving 88 participants were included. Sample sizes ranged from 16 to 23 participants. The included samples consisted of male or female participants and included adolescent athletes, young adult athletes, and middle-aged women.

All five studies used crossover designs in which the same participants completed multiple intervention conditions. The studies compared caffeine plus music with a control or placebo condition and with one or more single-component conditions. Four studies administered caffeine using a body-mass-adjusted dose: three studies used 3 mg/kg, and one study used 6 mg/kg. In contrast, Alsaeed et al. administered a fixed dose of 100 mg of caffeine in capsule form. Therefore, caffeine dosing was not adjusted to body mass in all included studies. The difference between fixed and body-mass-adjusted dosing was retained in the data extraction and should be considered when interpreting the between-study heterogeneity. Music interventions included preferred music, self-selected music, and warm-up music. Exercise protocols and assessment points varied substantially across studies (Table 1). The study-level paired effect estimates, paired standard errors, and corresponding sampling variances used in each analysis are provided in Supplementary Table S2. Estimates from the same crossover study were included in separate comparator-specific meta-analyses and were not treated as independent observations within a single pooled analysis.

### 3.3. Risk of Bias Assessment

The risk-of-bias assessment is presented in Figure 2. Most studies were judged to be at low risk of bias for random-sequence generation, incomplete outcome data, selective reporting, and outcome assessment. One study provided insufficient information regarding allocation concealment, and one study was judged to be at high risk of bias for participant and personnel blinding.

Because all studies used crossover designs, intervention order, washout periods, and potential period and carryover effects were also considered. Reporting of these design-specific features was incomplete in some studies. The risk-of-bias judgements were therefore considered when evaluating the certainty of the evidence.

The results emphasize the study-specific findings within each outcome and comparator contrast. Individual effect estimates and their confidence intervals are reported in Supplementary Table S2. The pooled estimates in Table 2 and Figure 3, Figure 4, Figure 5 and Figure 6 are presented as secondary exploratory summaries and should be interpreted alongside the variation among the constituent studies.

### 3.4. Effects of Caffeine Plus Music on Rating of Perceived Exertion

Study-specific RPE estimates differed substantially across the investigated conditions. For caffeine plus music versus placebo plus no music, Alsaeed et al. (2024) [17] reported an SMD of −4.05 (95% CI −5.32 to −2.79), and Delleli et al. (2023) [19] reported −3.20 (−4.28 to −2.11). The estimate in Delleli et al. (2024) [20] was −0.76 (−1.49 to −0.04), whereas those in Bougrine et al. (2025) [18] and Qiu et al. (2025) [21] were −0.05 (−0.73 to 0.62) and 0.40 (−0.18 to 0.99), respectively. Thus, the observed estimates included large reductions, smaller reductions, and estimates close to the null or in the opposite direction.

The secondary comparisons also showed substantial variation. Against caffeine alone, study-specific SMDs ranged from −4.66 to 0.12; against music alone, they ranged from −1.50 to 0.26. These ranges describe the observed point estimates and are not confidence or prediction intervals. The complete study-specific estimates and confidence intervals are provided in Table S2. This pattern does not establish a stable magnitude of RPE reduction across the investigated settings or a consistent incremental benefit over either component alone. The pooled summaries in Table 2 and Figure 3 should therefore not be interpreted as expected effects for a particular exercise population or protocol.

### 3.5. Effects of Caffeine Plus Music on Exercise Enjoyment

PACES data were available from three crossover studies, each involving 16 participants. The study-specific point estimates favoured greater enjoyment with caffeine plus music, but their magnitudes differed substantially in two comparisons. Against placebo plus no music, Alsaeed et al. (2024) [17] reported an SMD of 6.95 (95% CI 5.01 to 8.90), compared with 2.67 (1.69 to 3.66) in Delleli et al. (2023) [19] and 1.63 (0.82 to 2.45) in Delleli et al. (2024) [20]. Against caffeine alone, the corresponding estimates were 9.80 (7.14 to 12.46), 0.66 (−0.05 to 1.38), and 1.42 (0.63 to 2.20), respectively.

The estimates against music alone were more similar: 2.18 (1.28 to 3.07), 2.77 (1.77 to 3.77), and 2.35 (1.42 to 3.28), respectively. These comparisons involved the same three small samples and do not constitute evidence from additional independent populations. The descriptive findings identify a possible enjoyment-related benefit, but do not establish a reproducible magnitude or its practical importance. In particular, a single pooled value obscures the markedly different magnitudes observed against placebo plus no music and caffeine alone. The pooled results are therefore presented only as secondary exploratory summaries in Table 2 and Figure 4.

### 3.6. Effects of Caffeine Plus Music on Feeling Scale Scores

For Feeling Scale scores, the study-specific findings differed markedly. Against placebo plus no music, Delleli et al. (2023) [19] reported an SMD of 4.80 (95% CI 3.37 to 6.23), whereas Delleli et al. (2024) [20] and Qiu et al. (2025) [21] reported 0.49 (−0.22 to 1.19) and −0.25 (−0.83 to 0.33), respectively. Against caffeine alone, the two estimates were 3.15 (2.07 to 4.22) and 0.00 (−0.69 to 0.69). Against music alone, estimates ranged from −0.97 to 2.54, with effects in opposite directions across studies (Table S2). These findings do not establish a consistent response in affective valence. Secondary exploratory pooled summaries are provided in Table 2 and Figure 5.

### 3.7. Effects of Caffeine Plus Music on Heart Rate

Two studies provided heart-rate data. Against placebo plus no music, Delleli et al. (2024) [20] reported an SMD of −2.93 (95% CI −3.96 to −1.90), whereas Qiu et al. (2025) [21] reported 0.18 (−0.40 to 0.76). Against music alone, the corresponding estimates were −1.98 (−2.85 to −1.12) and 0.09 (−0.48 to 0.67). The contrast between these study-specific findings limits interpretation of a pooled average as an expected heart-rate response. Secondary exploratory summaries are reported in Table 2 and Figure 6. No pooled estimate was calculated against caffeine alone because fewer than two eligible study estimates were available.

A summary of the paired random-effects meta-analysis results across all outcomes and comparator conditions is presented in Table 2.

### 3.8. Additional Analyses

No subgroup analysis or meta-regression was conducted because only five independent studies were included, with fewer studies available for several outcomes. Formal analyses of small-study effects, including funnel plots, Egger-type tests, and trim-and-fill methods, were not conducted because every synthesis contained fewer than 10 independent studies. Publication bias could therefore not be statistically assessed and cannot be excluded.

### 3.9. Certainty of Evidence

The certainty of evidence for the primary comparison was assessed using the GRADE framework. The certainty was rated as very low for all four outcomes. Evidence for RPE and PACES was downgraded because of risk-of-bias concerns, very serious inconsistency, and serious imprecision. Evidence for FS and HR was downgraded because of risk-of-bias concerns, very serious inconsistency, and very serious imprecision. Publication bias could not be formally assessed because fewer than 10 independent studies were available for each outcome and therefore cannot be excluded (Table 3).

## 4. Discussion

This review identified five small crossover studies involving 88 participants. Its principal contribution is to describe the patterns and limitations of the available experimental evidence. The study-specific results do not establish a stable magnitude of benefit across the populations and exercise conditions investigated. RPE responses varied substantially, whereas PACES point estimates favoured the combined intervention but differed markedly in magnitude for comparisons with placebo plus no music and caffeine alone. Feeling Scale and heart-rate findings also varied across studies. These distinctions are more informative than a general statement that the combined intervention was effective or ineffective. However, statistical significance should not be equated with evidential certainty. Confidence intervals excluding the null indicate statistical significance under the assumptions of the fitted models, but do not resolve concerns about risk of bias, inconsistency, or imprecision. Only two to five small crossover studies contributed to each analysis, and all four primary analyses showed substantial heterogeneity, with I^2^ exceeding 90%. The GRADE assessment rated the certainty of evidence as very low for all primary outcomes. Accordingly, even the statistically significant primary findings for RPE and PACES should be regarded primarily as hypothesis-generating observations rather than confirmatory evidence of intervention effectiveness. These findings justify further investigation in adequately powered, methodologically rigorous studies but do not provide sufficient support for firm practical recommendations.

The appropriateness of quantitative pooling itself also requires consideration. The rationale for presenting an exploratory synthesis was that studies within each analysis addressed the same outcome construct and comparator-defined question, with the three comparator contrasts analysed separately. However, this shared framework does not establish that the participant populations, caffeine regimens, music interventions, and exercise protocols are sufficiently comparable to support a broadly meaningful average effect. With only two to five studies per analysis, both the pooled effects and estimates of between-study variability remain uncertain, and the averages may poorly represent individual study settings. Random-effects modelling accommodates variation in underlying effects but cannot resolve inadequate clinical comparability; describing an analysis as exploratory does not remove this limitation. The pooled estimates therefore provide only a limited numerical summary of the available experiments and should be interpreted alongside the study-specific estimates and experimental conditions. Their presentation should not be taken to imply that the evidence base is sufficiently mature to establish intervention effectiveness. The interpretability of the pooled averages remains uncertain, and no firm practical recommendations can be supported.

For RPE, averaging large reductions with estimates close to the null or in the opposite direction obscures uncertainty about whether a comparable reduction can be expected in another exercise setting [22]. For PACES, the central issue is different: the direction of the point estimates was consistent, but the size of the apparent benefit was not. The more similar PACES estimates against music alone remain observations from three small samples and do not establish broad reproducibility or clinical importance. Participant characteristics and intervention conditions also differed; for example, the fixed 100 mg caffeine regimen in the sample of middle-aged women differed from the body-mass-adjusted regimens used in athletic samples [11,23]. These contextual differences cannot be separated sufficiently to identify which population or intervention characteristics explain the observed responses.

The unusually large, pooled PACES effects (SMDs = 2.41–3.56) warrant additional scrutiny. These estimates were based on three studies with only 16 participants each, making both mean differences and estimates of variability susceptible to sampling fluctuations. Although a small-sample correction was applied, this does not eliminate sampling uncertainty or study-level biases. An SMD depends on both the mean difference and the standard deviation used for standardization; a smaller standard deviation can produce a larger SMD for the same absolute score difference. Differences in outcome assessment procedures and timing, exercise context, and sample characteristics may therefore contribute to the observed standardized magnitudes. Moreover, awareness of music exposure in comparisons with no music may influence self-reported enjoyment through expectancy effects. These are plausible methodological contributions, but their extent cannot be established from the available evidence.

Statistical magnitude should therefore not be equated with clinical or practical importance. The present review did not establish whether the observed differences exceeded a context-appropriate threshold for meaningful improvement on the original PACES scale or translated into sustained exercise participation. Accordingly, these large, standardized estimates should be interpreted as exploratory signals of possible improvements in enjoyment, with uncertain practical relevance, rather than evidence of an exceptionally strong and broadly applicable intervention effect.

The retained random-effects analyses estimate averages across potentially different effects; they do not establish an effect shared across populations or guarantee that the average represents any exercise setting [7,24]. Their role is therefore limited to secondary numerical summaries of the analyses undertaken. The review’s conclusions are based primarily on the study-specific findings, their uncertainty, and the very low certainty of evidence. The practical implication is that the current literature identifies questions requiring replication, including whether RPE reductions are reproducible and whether enjoyment improvements are meaningful under clearly specified conditions [25]. It does not identify a dependable, expected benefit or an optimal caffeine–music protocol. Descriptive synthesis does not remove the underlying limitations but makes their consequences for interpretation more explicit.

Several clinical and methodological differences may have contributed to the substantial heterogeneity. Caffeine protocols included a fixed dose of 100 mg and body-mass-adjusted doses of 3 or 6 mg/kg. These regimens provide different relative exposures and may therefore produce different physiological and perceptual responses. Likewise, preferred or self-selected music does not necessarily represent a standardized stimulus: differences in musical characteristics, motivational value, and exposure timing may influence attention, arousal, and enjoyment. Music used during warm-up may also have effects that depend on how long these responses persist into the subsequent exercise task. Differences in exercise modality and relative intensity may alter physiological demands and the extent to which music can distract attention from exertional sensations, while assessment timing may capture different stages of fatigue or recovery. Participant sex, age, and training status may further influence intervention responsiveness and the relative demands of the exercise task. These differences could therefore contribute to variation in both subjective outcomes and heart-rate responses.

These explanations remain tentative. The relevant characteristics varied together across studies, and only five independent studies were available, preventing reliable separation of their individual contributions through subgroup analysis or meta-regression. They should therefore be regarded as plausible sources of heterogeneity rather than established moderators.

The implications extend beyond identifying possible sources of variability. Across the four primary outcomes, I^2^ ranged from 91.9% to 96.2%, indicating substantial inconsistency and limiting confidence that a single pooled value adequately represents the different study settings. Although random-effects models allow underlying effects to vary, they do not resolve clinical or methodological differences. Their pooled estimates describe an average effect, and the corresponding 95% confidence intervals express uncertainty around that average rather than the range of effects across populations or exercise settings. Consequently, the apparent benefits for RPE and PACES cannot be assumed to occur consistently under different conditions. Given the substantial heterogeneity, small evidence base, and very low certainty of evidence, the pooled estimates should be interpreted as exploratory summaries rather than precise, broadly generalizable estimates of intervention effectiveness.

The simple comparison of caffeine plus music with a control condition estimates the overall effect of the combined intervention but does not formally demonstrate an additive or synergistic interaction between caffeine and music. Caffeine has been proposed to influence adenosine signaling, alertness, and perceived effort, whereas music may affect attentional focus, motivation, and affective responses [26]. However, the included studies were not designed to test these mechanisms and did not directly assess the relevant physiological or psychological mediators. These pathways should therefore be regarded only as possible explanations. Establishing synergy would require a factorial interaction contrast and its within-participant sampling variance; consequently, the present findings should not be interpreted as evidence that caffeine and music act synergistically.

This review has several limitations. Several limitations should be considered. First, only five crossover studies involving 88 participants were included, with two to five studies contributing to each analysis. The pooled estimates were therefore imprecise, and I^2^ exceeded 90% in all primary comparisons. The studies also differed substantially in participant age, sex, training status, caffeine dose, music selection, exercise protocol, and assessment timing. Although random-effects models and standardized mean differences were used, these methods do not explain or remove the underlying clinical heterogeneity. The pooled estimates should therefore be interpreted as exploratory averages rather than effects that are generalizable across populations and exercise settings.

Second, reporting of crossover-specific features, including intervention order, washout periods, period effects, and potential carryover effects, was incomplete in some studies. Some paired effect estimates and sampling variances also had to be reconstructed from the available statistics, making their reliability dependent on the completeness of the original reports. Although comparator-specific meta-analyses avoided including correlated estimates from the same trial within a single model, estimates across different comparator analyses remained correlated. No multiplicity adjustment was applied; therefore, the secondary comparisons and their pointwise confidence intervals should be interpreted cautiously.

Finally, the small number of studies precluded reliable sensitivity analysis, subgroup analysis, and meta-regression. Participant sex, caffeine dose, music type, and exercise modality could therefore be considered only as possible sources of heterogeneity rather than confirmed moderators. Formal assessment of small-study effects and publication bias was also not appropriate, and publication bias cannot be excluded. The certainty of evidence was very low for all primary outcomes because of risk-of-bias concerns, inconsistency, and imprecision. Accordingly, statistical significance should not be equated with confidence in the consistency or generalizability of the findings, which remain preliminary and hypothesis-generating.

The certainty of evidence was rated as very low for all four primary outcomes. For RPE, although the confidence interval narrowly excluded the null, confidence in the estimate was substantially limited by risk-of-bias concerns, very serious inconsistency, and serious imprecision. The evidence for PACES was also of very low certainty because it was based on only three studies and showed considerable heterogeneity and a wide confidence interval. For Feeling Scale scores, the confidence interval included effects in both directions, and the evidence was downgraded for risk of bias, very serious inconsistency, and very serious imprecision. The heart-rate evidence was similarly of very low certainty because only two studies contributed to the analysis and the estimate was highly heterogeneous and imprecise. Therefore, none of the primary outcomes provide moderate- or high-certainty evidence of an effect, and further studies may materially change both the magnitude and direction of the pooled estimates.

External validity is also limited by the small, specific samples, which included adolescent and young adult athletes and middle-aged women. The inclusion of different age groups, sexes, and athletic backgrounds should not be interpreted as sufficient evidence of comparable effects across these populations. Furthermore, the pooled estimates summarize responses to the particular caffeine dosing regimens, music selections and exposure schedules, and exercise protocols investigated. It remains uncertain whether similar effects would occur with different doses, non-preferred music, or other exercise modalities and intensities. Outcomes obtained under controlled experimental conditions also do not establish sustained benefits during habitual exercise or longer-term training. Accordingly, the applicability of these findings beyond the populations and conditions studied remains uncertain, and the current evidence is insufficient to support universal practical recommendations across exercise settings and populations.

## 5. Conclusions

In this exploratory meta-analysis of five small and clinically heterogeneous crossover studies, the pooled estimates suggested possible reductions in perceived exertion and increases in exercise enjoyment when caffeine was combined with music compared with placebo plus no music. However, the evidence was very uncertain and did not consistently demonstrate an incremental benefit over caffeine or music alone. No clear differences were observed for affective valence or heart rate. Given the very small evidence base, substantial heterogeneity, imprecision, and very low certainty of evidence, these findings should not be considered confirmatory or used to support firm practical recommendations. Further adequately powered and methodologically rigorous studies using more comparable populations, interventions, and outcome assessments are required.

## Figures and Tables

**Figure 1:**
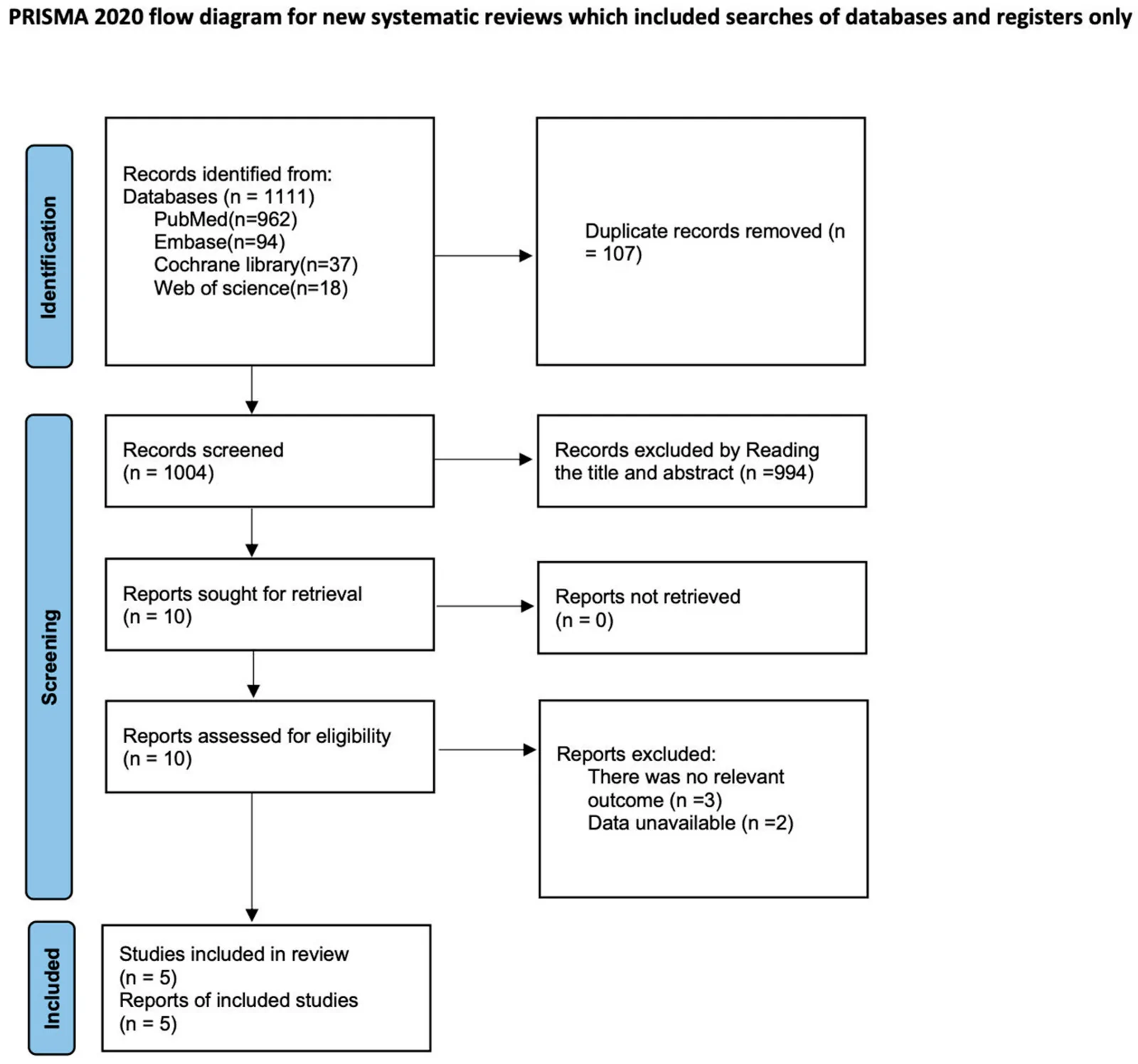
PRISMA flow diagram of study selection process.

**Figure 2:**
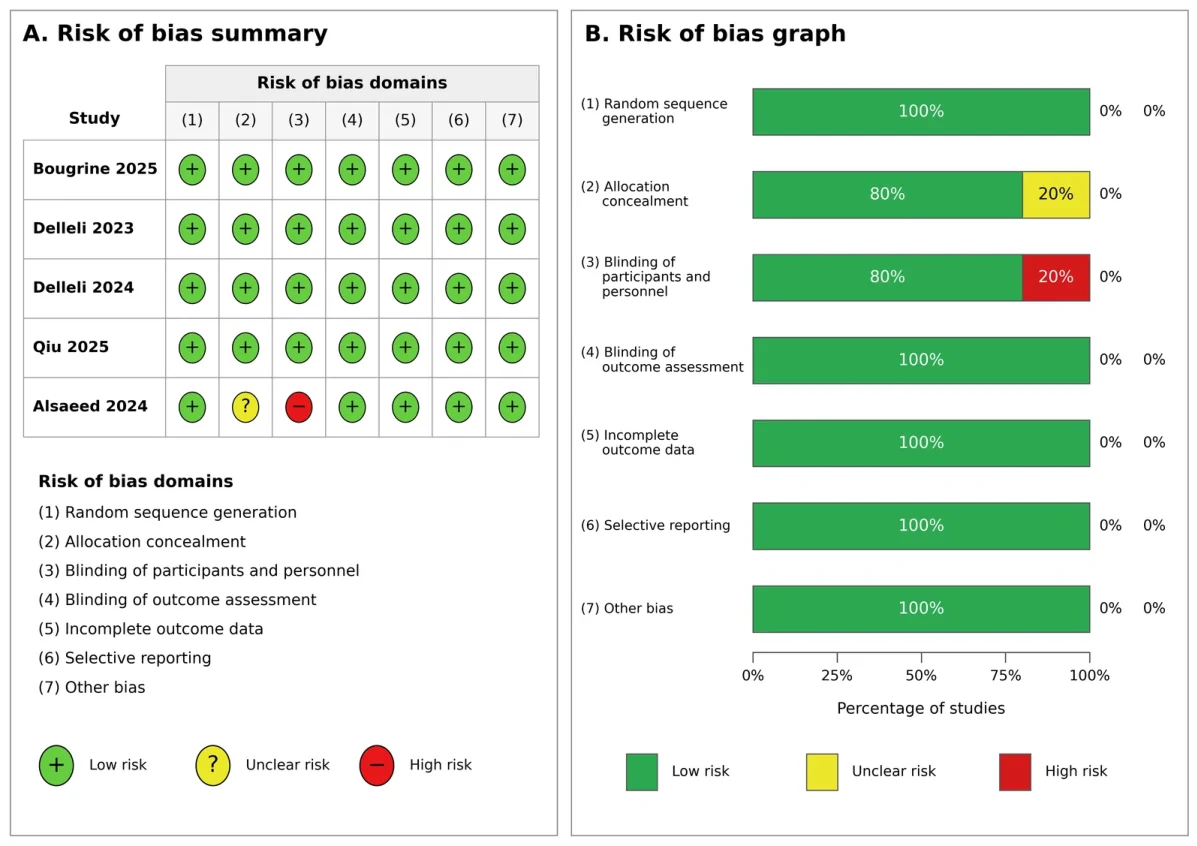
Risk of bias assessment of included studies [17,18,19,20,21].

**Figure 3:**
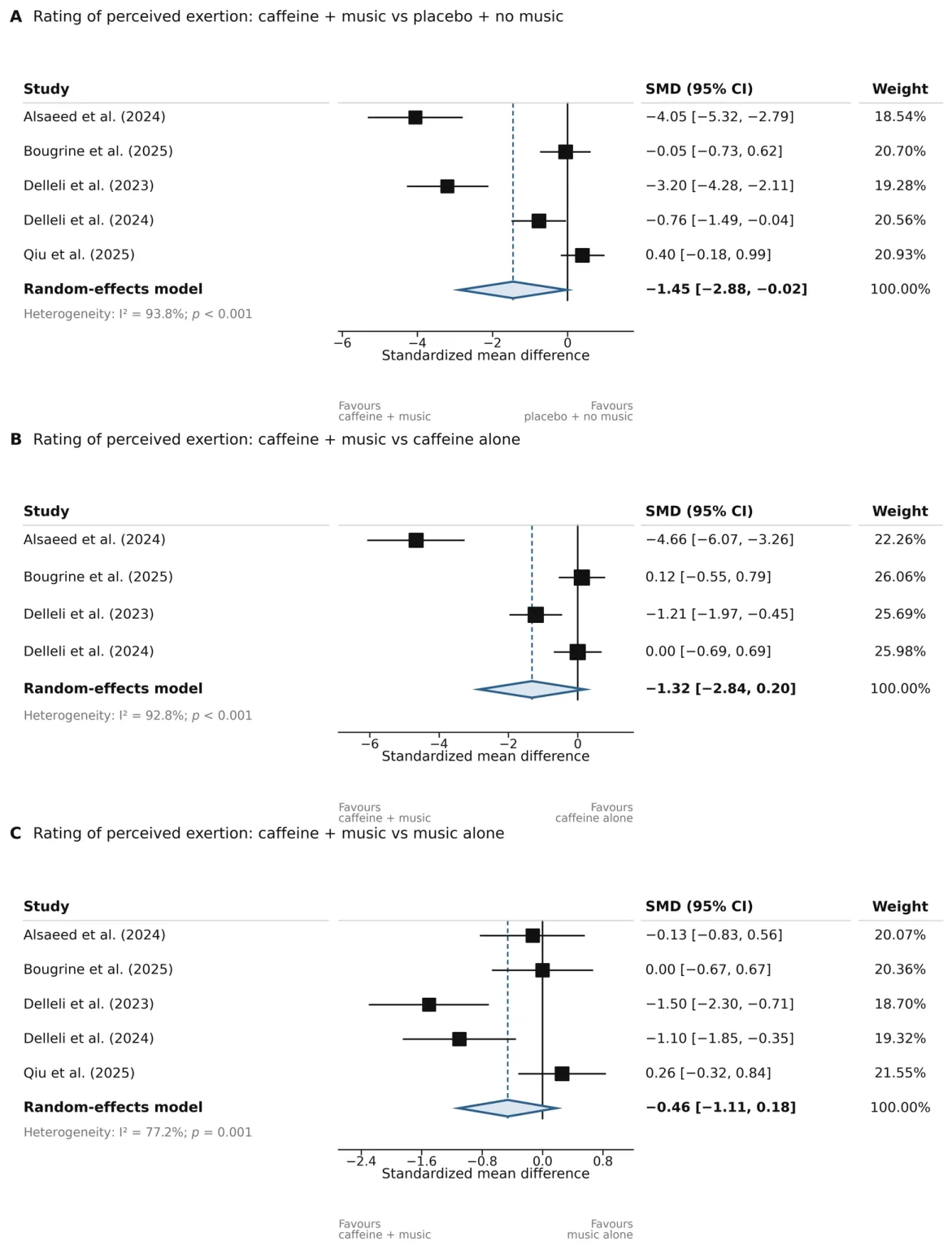
Random-effects meta-analyses of rating of perceived exertion for caffeine plus music versus (**A**) placebo plus no music, (**B**) caffeine alone, and (**C**) music alone [17,18,19,20,21].

**Figure 4:**
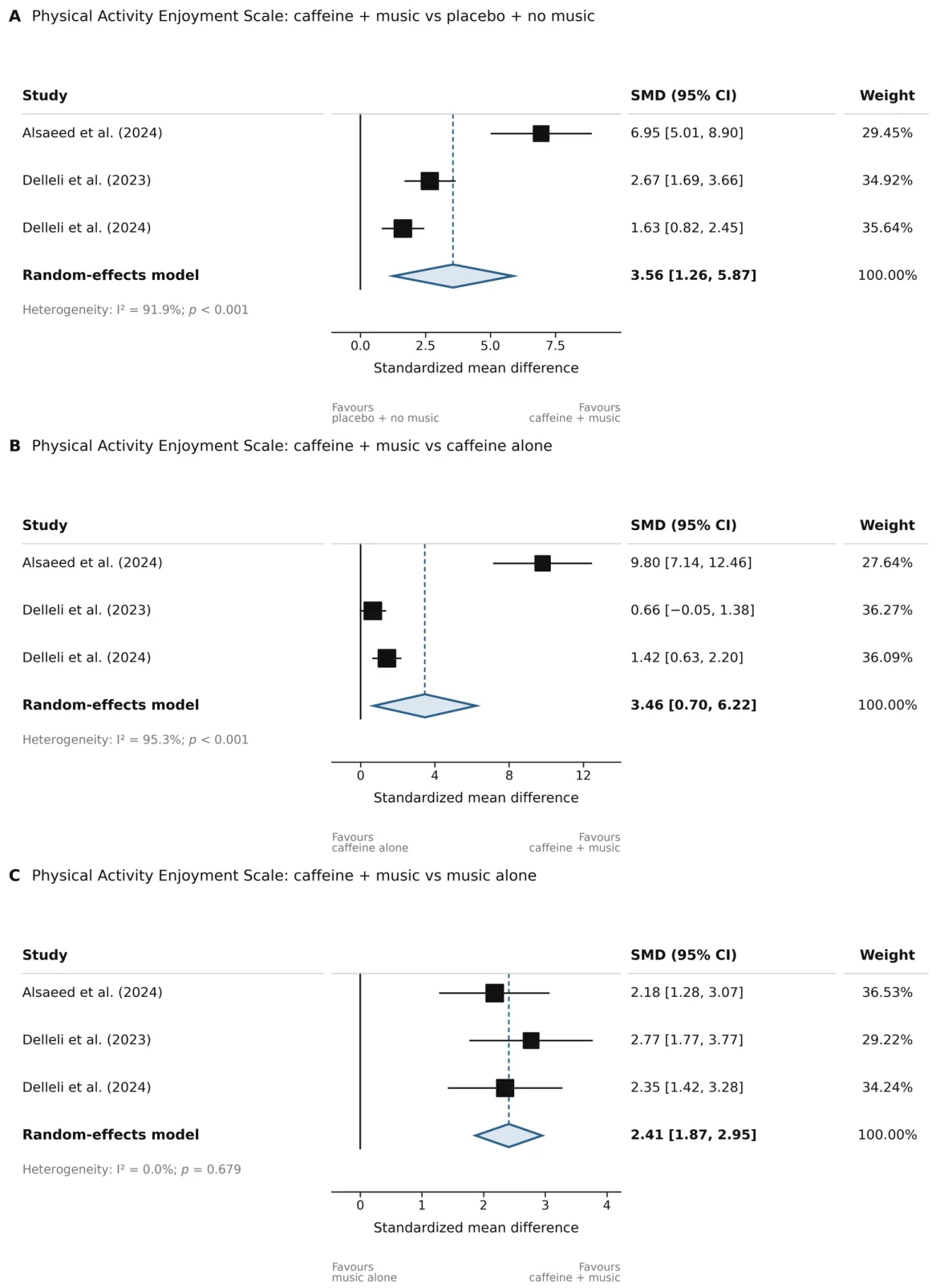
Random-effects meta-analyses of Physical Activity Enjoyment Scale scores for caffeine plus music versus (**A**) placebo plus no music, (**B**) caffeine alone, and (**C**) music alone [17,19,20].

**Figure 5:**
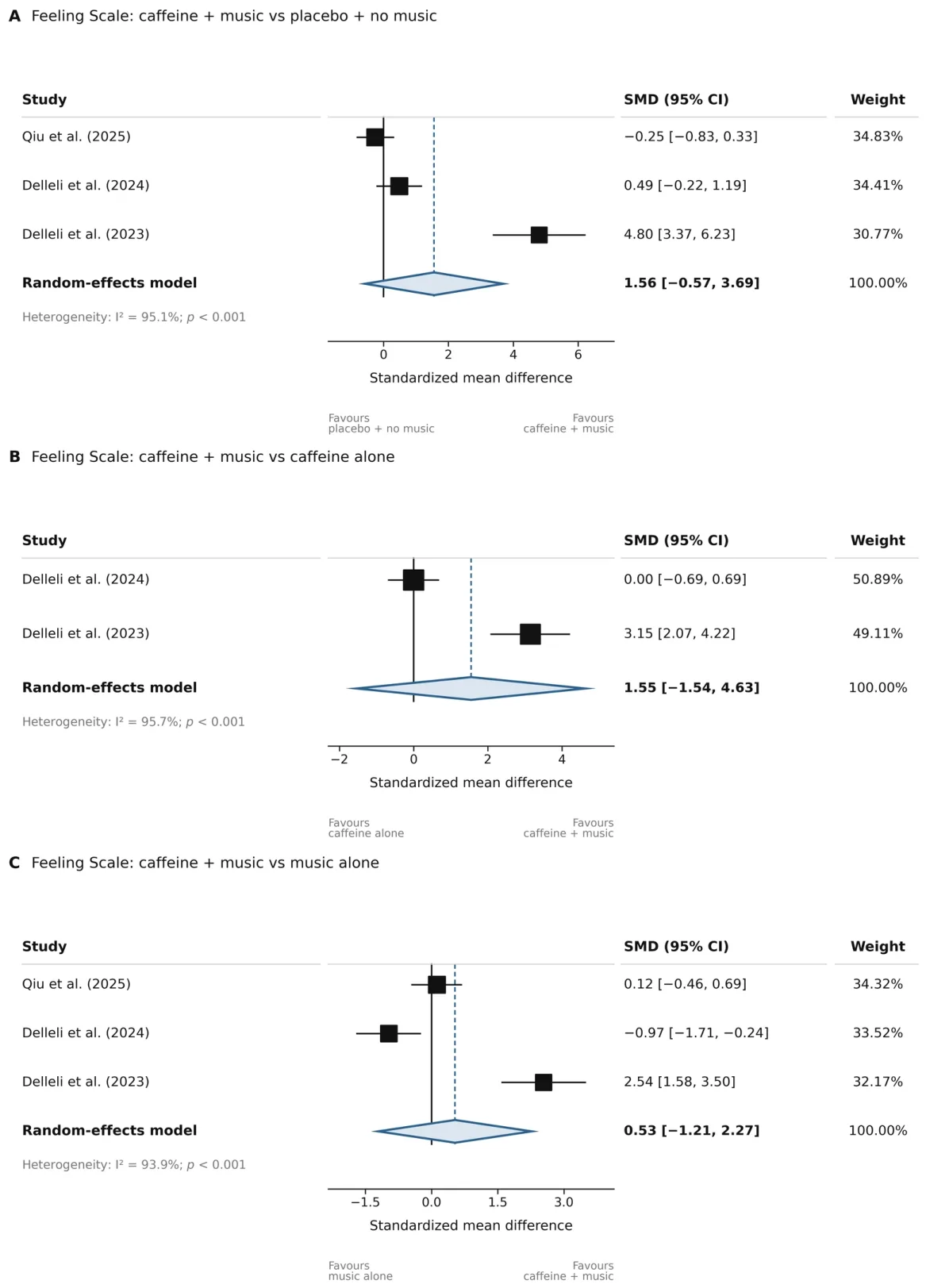
Random-effects meta-analyses of Feeling Scale scores for caffeine plus music versus (**A**) placebo plus no music, (**B**) caffeine alone, and (**C**) music alone [19,20,21].

**Figure 6:**
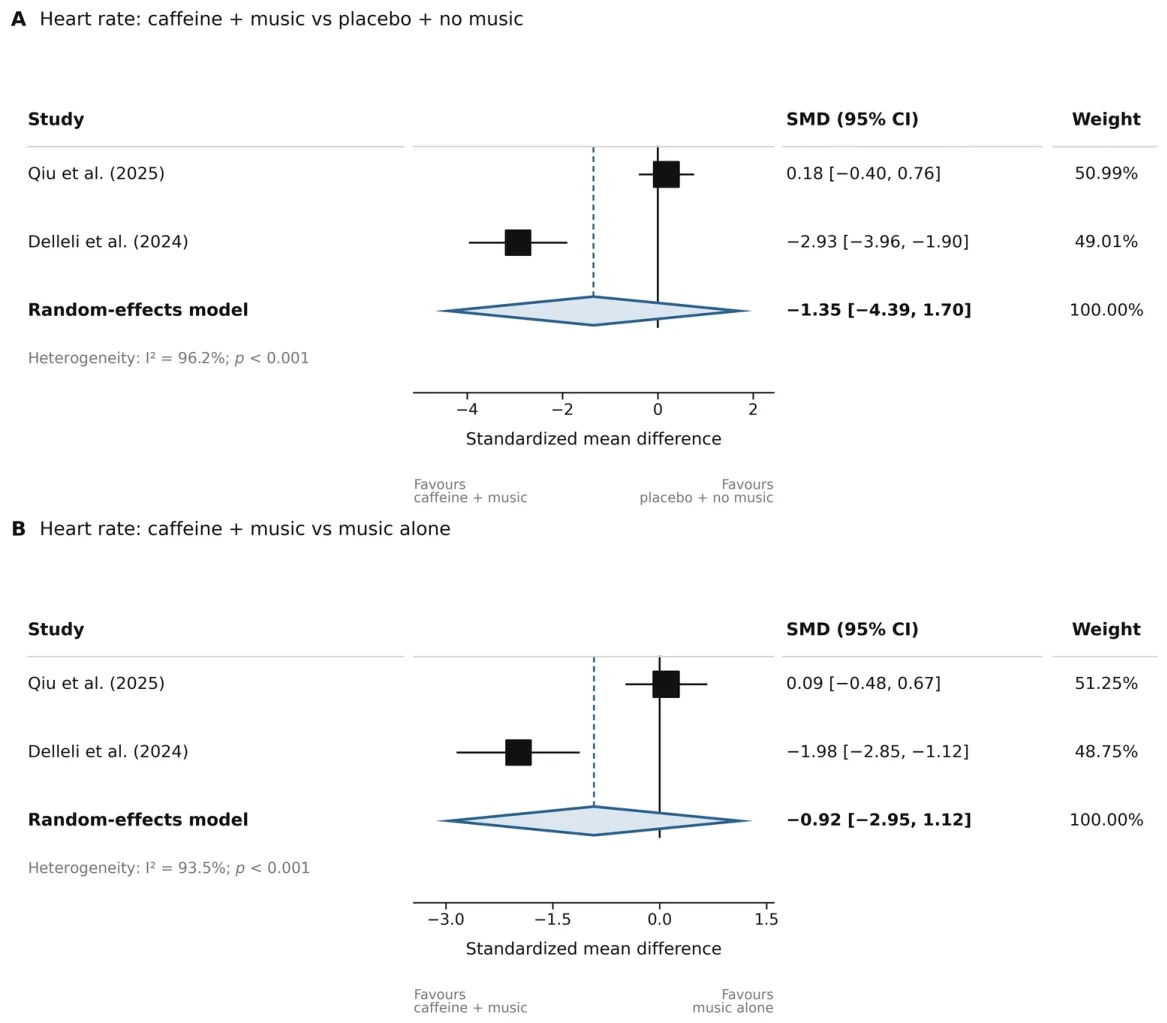
Random-effects meta-analyses of heart rate for caffeine plus music versus (**A**) placebo plus no music and (**B**) music alone [20,21].

**Table 1:** Characteristics of included studies.

Study	Year	Country	Sample Size	Age	Sex	Study Design	Combined Intervention	Eligible Comparators	Caffeine Dose	Music Intervention	Outcomes
Alsaeed et al. [17]	2024	Saudi Arabia	16	57 ± 1.8 years	Female	Single-blind crossover	Caffeine plus music	Control; caffeine alone; music alone	100 mg fixed dose; not adjusted to body mass	Preferred music	RPE, PACES
Bougrine et al. [18]	2025	France	17	16.7 ± 0.4 years	Female	Randomized double-blind crossover	Caffeine plus music	Control; caffeine alone; music alone	6 mg/kg body mass	Self-selected motivational music	RPE
Delleli et al. [19]	2023	Tunisia	16	18.25 ± 0.75 years	Male	Randomized double-blind counterbalanced crossover	Caffeine plus music	Placebo plus no music; caffeine alone; music alone	3 mg/kg body mass	Preferred warm-up music	RPE, PACES, FS
Delleli et al. [20]	2024	Tunisia	16	16–18 years	Female	Randomized double-blind counterbalanced crossover	Caffeine plus music	Placebo plus no music; caffeine alone; music alone	3 mg/kg body mass	Preferred warm-up music	RPE, PACES, FS, HR
Qiu et al. [21]	2025	China	23	21.0 ± 1.6 years	Male	Randomized double-blind crossover	Caffeine plus music	Placebo plus no music; music alone	3 mg/kg body mass	Self-selected warm-up music	RPE, FS, HR

Abbreviations: FS, Feeling Scale; HR, heart rate; PACES, Physical Activity Enjoyment Scale; RPE, rating of perceived exertion.

**Table 2:** Secondary exploratory random-effects summaries by outcome and comparator.

Outcome	Comparison	Studies, k	Paired SMD	95% CI	I^2^	Interpretation
RPE	CAF + music vs. placebo + no music	5	−1.45	−2.88 to −0.02	93.80%	Possible reduction; very heterogeneous
RPE	CAF + music vs. caffeine alone	4	−1.32	−2.84 to 0.20	92.80%	No clear difference
RPE	CAF + music vs. music alone	5	−0.46	−1.11 to 0.18	77.20%	No clear difference
PACES	CAF + music vs. placebo + no music	3	3.56	1.26 to 5.87	91.90%	Possible increase; very heterogeneous
PACES	CAF + music vs caffeine alone	3	3.46	0.70 to 6.22	95.30%	Possible increase; very heterogeneous
PACES	CAF + music vs. music alone	3	2.41	1.87 to 2.95	0.00%	Possible increase; only three studies
FS	CAF + music vs. placebo + no music	3	1.56	−0.57 to 3.69	95.10%	No clear difference
FS	CAF + music vs. caffeine alone	2	1.55	−1.54 to 4.63	95.70%	No clear difference
FS	CAF + music vs. music alone	3	0.53	−1.21 to 2.27	93.90%	No clear difference
HR	CAF + music vs. placebo + no music	2	−1.35	−4.39 to 1.70	96.20%	No clear difference
HR	CAF + music vs. music alone	2	−0.92	−2.95 to 1.12	93.50%	No clear difference

Effect estimates and sampling variances were calculated using within-participant paired data. CI, confidence interval; CAF, caffeine; FS, Feeling Scale; HR, heart rate; PACES, Physical Activity Enjoyment Scale; RPE, rating of perceived exertion; SMD, standardized mean difference.

**Table 3:** GRADE Summary of Findings for caffeine plus music versus placebo plus no music.

Outcome	Studies	Participants	Paired Effect Estimate	Heterogeneity	Certainty	Interpretation
RPE	5	88	SMD −1.45 (95% CI −2.88 to −0.02)	I^2^ = 93.8%	⊕◯◯◯ Very low	Possible reduction, but confidence is very limited
PACES	3	48	SMD 3.56 (95% CI 1.26 to 5.87)	I^2^ = 91.9%	⊕◯◯◯ Very low	Possible increase, but confidence is very limited
FS	3	55	SMD 1.56 (95% CI −0.57 to 3.69)	I^2^ = 95.1%	⊕◯◯◯ Very low	No clear evidence of a difference
HR	2	39	SMD −1.35 (95% CI −4.39 to 1.70)	I^2^ = 96.2%	⊕◯◯◯ Very low	No clear evidence of a difference

Certainty was downgraded because of the very small number of studies and participants, serious or very serious inconsistency, and serious imprecision. CI, confidence interval; FS, Feeling Scale; HR, heart rate; PACES, Physical Activity Enjoyment Scale; RPE, rating of perceived exertion; SMD, standardized mean difference.

## Data Availability

The data supporting the findings of this study are included in the article and its Supplementary Materials. Further inquiries may be directed to the corresponding author.

## Supplementary Materials

The following supporting information can be downloaded at: https://www.mdpi.com/article/10.3390/nu18203441/s1, Table S1: Electronic database search strategies and search results; Table S2: Study-level paired standardized mean differences and sampling variances used in the meta-analyses.

## Abbreviations

The following abbreviations are used in this manuscript:

CAF	Caffeine
CI	Confidence interval
CON	Control condition
FS	Felt Scale scores
GRADE	Grading of Recommendations Assessment, Development and Evaluation
HR	Heart rate
PACES	Physical Activity Enjoyment Scale
PRISMA	Preferred Reporting Items for Systematic Reviews and Meta-Analyses
RPE	Rating of Perceived Exertion
SD	Standard deviation
SE	Standard error
SMD	Standardized mean difference

## References

[1] Cheng M., Ni J., Liu F., Wang S. (2026). Optimal type and dose of exercise to improve fall behavior in older adults: A systematic evaluation and network meta-analysis. Ageing Res. Rev..

[2] Pellegrini C.A., Carlesso L.C., White D.K. (2026). More than a prescription: The need for behavioral theory to guide exercise interventions for osteoarthritis—A narrative review. Osteoarthr. Cartil..

[3] Balbim G.M., Boa Sorte Silva N.C., Falck R.S., Kramer A.F., Voss M.W., Liu-Ambrose T. (2025). 24-hour activity cycle behaviors and gray matter volume in mild cognitive impairment. Alzheimers Dement..

[4] Buyck C., Delpire B., Dausin C., Vermunicht P., Desteghe L., Bekhuis Y., Pauwels R., de Paepe J., Ruiz-Carmona S., La Gerche A. (2026). High heart rate peaks from chest-strap recordings in athletes: Prevalence, characteristics, and clinical relevance. Eur. J. Prev. Cardiol..

[5] Di Gioia G., Squeo M.R., Ferrera A., Spera F.R., Maestrini V., Monosilio S., Mango F., Paoletti G., Serdoz A., Bernardi M. (2026). Athlete’s Heart or Heart at Risk? Cardiac Remodeling and Exercise-Induced Ventricular Arrhythmias in Elite Athletes. Circ. Arrhythm. Electrophysiol..

[6] Naderi A., Rothschild J.A., Santos H.O., Hamidvand A., Koozehchian M.S., Ghazzagh A., Berjisian E., Podlogar T. (2025). Nutritional Strategies to Improve Post-exercise Recovery and Subsequent Exercise Performance: A Narrative Review. Sports Med..

[7] Silva H., Del Coso J., Pickering C. (2025). Caffeine and Sports Performance: The Conflict between Caffeine Intake to Enhance Performance and Avoiding Caffeine to Ensure Sleep Quality. Sports Med..

[8] Tang J., Xu Z., Wang F., Guan L., Qi B., Zou Y. (2025). Caffeine Inhibits Tau Aggregation and Destabilizes the Fibril Associated with Chronic Traumatic Encephalopathy: A REST2 and Conventional MD Simulations Study. J. Chem. Inf. Model..

[9] Hori K., Wee C.P., Liu N., Parsons J., Choi J.S. (2026). Lower Music Volume Levels in Fitness Class and Perceived Exercise Intensity. JAMA Otolaryngol. Head Neck Surg..

[10] Stylianou K., Zamboglou C., Nicolaou A., Contopoulos G., Lopez G. (2025). Symphony of Strength: Music and Integrative Oncology in the Management of Glioblastoma. Curr. Psychiatry Rep..

[11] Kerksick C.M., Arent S., Schoenfeld B.J., Stout J.R., Campbell B., Wilborn C.D., Taylor L., Kalman D., Smith-Ryan A.E., Kreider R.B. (2017). International society of sports nutrition position stand: Nutrient timing. J. Int. Soc. Sports Nutr..

[12] Jebabli N., Dhahbi W., Boujabli M., Khlifi M., Ouerghi N., Bouassida A., Ben Abderrahman A., Tillaar R.v.D. (2026). Effects of the single and combined effect of music and other strategies on combat sport performance: A systematic review and meta-analysis. Front. Sports Act. Living.

[13] Bittencourt de Santa Ritta G., Brito Soares L.M., Abreu A.C.P., Mendes G.F., Saunders B., Reis C.E.G. (2026). Addressing the Evidence Gap: Ergogenic Effects of Caffeine on Cycling Performance in Females-A Systematic Review and Three-Level Meta-Analysis. Int. J. Sport Nutr. Exerc. Metab..

[14] Haddaway N.R., Page M.J., Pritchard C.C., McGuinness L.A. (2022). PRISMA2020: An R package and Shiny app for producing PRISMA 2020-compliant flow diagrams, with interactivity for optimised digital transparency and Open Synthesis. Campbell Syst. Rev..

[15] Higgins J.P., Altman D.G., Gøtzsche P.C., Jüni P., Moher D., Oxman A.D., Savović J., Schulz K.F., Weeks L., Sterne J.A.C. (2011). The Cochrane Collaboration’s tool for assessing risk of bias in randomised trials. BMJ.

[16] Guyatt G., Oxman A.D., Akl E.A., Kunz R., Vist G., Brozek J., Norris S., Falck-Ytter Y., Glasziou P., DeBeer H. (2011). GRADE guidelines: 1. Introduction-GRADE evidence profiles and summary of findings tables. J. Clin. Epidemiol..

[17] Alsaeed M.I., Waer F.B. (2024). Music and Caffeine Intake Effects on Gait, and Its Relationship with Psychological Parameters, in Middle-Aged Women. J. Multidiscip. Healthc..

[18] Bougrine H., Paillard T., Jebabli N., Ceylan H., Maitre J., Dergaa I., Stefanica V., Ben Abderrahman A. (2025). Ergogenic Effects of Combined Caffeine Supplementation and Motivational Music on Anaerobic Performance in Female Handball Players: A Randomized Double-Blind Controlled Trial. Nutrients.

[19] Delleli S., Ouergui I., Messaoudi H., Ballmann C., Ardigò L., Chtourou H. (2023). Effects of caffeine consumption combined with listening to music during warm-up on taekwondo physical performance, perceived exertion and psychological aspects. PLoS ONE.

[20] Delleli S., Ouergui I., Messaoudi H., Bridge C., Ardigò L., Chtourou H. (2024). Warm-up music and low-dose caffeine enhance the activity profile and psychophysiological responses during simulated combat in female taekwondo athletes. Sci. Rep..

[21] Qiu B., Wang Z., Zhang Y., Cui Y., Diao P., Liu K., Del Coso J., Liu C. (2025). Effects of Caffeine Intake Combined with Self-Selected Music During Warm-Up on Anaerobic Performance: A Randomized, Double-Blind, Crossover Study. Nutrients.

[22] Guest N.S., VanDusseldorp T.A., Nelson M.T., Grgic J., Schoenfeld B.J., Jenkins N.D.M., Arent S.M., Antonio J., Stout J.R., Trexler E.T. (2021). International society of sports nutrition position stand: Caffeine and exercise performance. J. Int. Soc. Sports Nutr..

[23] Grgic J., Pedisic Z., Saunders B., Artioli G.G., Schoenfeld B.J., McKenna M.J., Bishop D.J., Kreider R.B., Stout J.R., Kalman D.S. (2021). International Society of Sports Nutrition position stand: Sodium bicarbonate and exercise performance. J. Int. Soc. Sports Nutr..

[24] Grgic J. (2021). Effects of Caffeine on Resistance Exercise: A Review of Recent Research. Sports Med..

[25] Chia J.S., Barrett L.A., Chow J.Y., Burns S.F. (2017). Effects of Caffeine Supplementation on Performance in Ball Games. Sports Med..

[26] Grgic J., Mikulic P., Schoenfeld B.J., Bishop D.J., Pedisic Z. (2019). The Influence of Caffeine Supplementation on Resistance Exercise: A Review. Sports Med..

