# Supplementary material for: Effects of Combined Music and Caffeine on Psychophysiological Responses During Exercise: A Systematic Review and Exploratory Meta-Analysis
Source: Nutrients. 2026 Oct 20;18(20):3441. doi: 10.3390/nu18203441 (PMC13708094; doi:10.3390/nu18203441)
Supplement: Supplementary file 1 [file nutrients-18-03441-s001.zip › nutrients-4506702-supplementary.pdf]

Supplementary Material

Table S1. Electronic database search strategies and search results

Database and platform	Coverage	Reconstructed search strategy*	Restrictions	Records retrieved
PubMed (National Library of Medicine)	Database inception to search date	("Caffeine"[Mesh] OR caffeine[tiab] OR coffee[tiab]) AND ("Music"[Mesh] OR music*[tiab] OR "auditory stimulation"[tiab] OR "rhythmic auditory stimulation"[tiab]) AND ("Exercise"[Mesh] OR exercis*[tiab] OR "physical activity"[tiab] OR "physical activities"[tiab] OR sport*[tiab] OR train*[tiab]) AND (psychophysiolog*[tiab] OR "perceived exertion"[tiab]OR RPE[tiab] OR enjoyment[tiab] OR "physical activity enjoyment scale"[tiab] OR PACES[tiab] OR "feeling scale"[tiab] OR affect*[tiab] OR arousal[tiab] OR "heart rate"[tiab])	No language, publication-status, or date restrictions	962
Embase (interface used to be verified from the original search log)	Database inception to search date	('caffeine'/exp OR caffeine:ti,ab,kw OR coffee:ti,ab,kw) AND ('music'/exp OR music*:ti,ab,kwOR 'auditory stimulation':ti,ab,kw OR 'rhythmic auditory stimulation':ti,ab,kw) AND ('exercise'/expOR 'physical activity'/exp OR exercis*:ti,ab,kw OR 'physical activity':ti,ab,kw OR sport*:ti,ab,kw OR train*:ti,ab,kw) AND ('psychophysiology'/exp OR psychophysiolog*:ti,ab,kw OR 'perceived exertion':ti,ab,kw OR rpe:ti,ab,kw OR enjoyment:ti,ab,kw OR 'physical activity enjoyment scale':ti,ab,kw OR paces:ti,ab,kw OR 'feeling scale':ti,ab,kw OR affect*:ti,ab,kw OR arousal:ti,ab,kw OR 'heart rate':ti,ab,kw)	No language, publication-status, or date restrictions	94
Cochrane Library (Wiley)	Database inception to search date	([mh Caffeine] OR caffeine:ti,ab,kw OR coffee:ti,ab,kw) AND ([mh Music] OR music*:ti,ab,kwOR "auditory stimulation":ti,ab,kw OR "rhythmic auditory stimulation":ti,ab,kw) AND ([mh Exercise] OR exercis*:ti,ab,kw OR "physical activity":ti,ab,kw OR sport*:ti,ab,kw OR train*:ti,ab,kw) AND (psychophysiolog*:ti,ab,kw OR "perceived exertion":ti,ab,kw OR RPE:ti,ab,kw OR enjoyment:ti,ab,kw OR PACES:ti,ab,kw OR "feeling scale":ti,ab,kw OR affect*:ti,ab,kw OR arousal:ti,ab,kw OR "heart rate":ti,ab,kw)	No language, publication-status, or date restrictions	37
Web of Science Core Collection	Database inception to search date	TS=((caffeine OR coffee) AND (music* OR "auditory stimulation" OR "rhythmic auditory stimulation") AND (exercis* OR "physical activity" OR sport* OR train*) AND (psychophysiolog* OR "perceived exertion" OR RPE OR enjoyment OR "physical activity enjoyment scale" OR	No language, publication-status,	18

(Clarivate)		PACES OR "feeling scale" OR affect* OR arousal OR "heart rate"))	or date restrictions	
Total	—	—	—	1111

Table S2. Study-level paired standardized mean differences and sampling variances used in the meta-analyses.

Comparison	Study	Paired n*	Paired SMD	95% CI	Paired SE†	Sampling variance
Caffeine + music vs placebo + no music	Alsaeed et al. (2024)	16	−4.05	−5.32 to −2.79	0.645	0.4166
Caffeine + music vs placebo + no music	Bougrine et al. (2025)	17	−0.05	−0.73 to 0.62	0.344	0.1186
Caffeine + music vs placebo + no music	Delleli et al. (2023)	16	−3.20	−4.28 to −2.11	0.554	0.3064
Caffeine + music vs placebo + no music	Delleli et al. (2024)	16	−0.76	−1.49 to −0.04	0.37	0.1368
Caffeine + music vs placebo + no music	Qiu et al. (2025)	23	0.4	−0.18 to 0.99	0.298	0.0891
Caffeine + music vs caffeine alone	Alsaeed et al. (2024)	16	−4.66	−6.07 to −3.26	0.717	0.5139
Caffeine + music vs caffeine alone	Bougrine et al. (2025)	17	0.12	−0.55 to 0.79	0.342	0.1169
Caffeine + music vs caffeine alone	Delleli et al. (2023)	16	−1.21	−1.97 to −0.45	0.388	0.1504
Caffeine + music vs caffeine alone	Delleli et al. (2024)	16	0	−0.69 to 0.69	0.352	0.1239
Caffeine + music vs music alone	Alsaeed et al. (2024)	16	−0.13	−0.83 to 0.56	0.355	0.1257
Caffeine + music vs music alone	Bougrine et al. (2025)	17	0	−0.67 to 0.67	0.342	0.1169
Caffeine + music vs music alone	Delleli et al. (2023)	16	−1.50	−2.30 to −0.71	0.406	0.1645
Caffeine + music vs music alone	Delleli et al. (2024)	16	−1.10	−1.85 to −0.35	0.383	0.1464
Caffeine + music vs music alone	Qiu et al. (2025)	23	0.26	−0.32 to 0.84	0.296	0.0876
Comparison	Study	Paired n*	Paired SMD	95% CI	Paired SE†	Sampling variance
Caffeine + music vs placebo + no music	Alsaeed et al. (2024)	16	6.95	5.01 to 8.90	0.992	0.9848
Caffeine + music vs placebo + no music	Delleli et al. (2023)	16	2.67	1.69 to 3.66	0.503	0.2526
Caffeine + music vs placebo + no music	Delleli et al. (2024)	16	1.63	0.82 to 2.45	0.416	0.1729
Caffeine + music vs caffeine alone	Alsaeed et al. (2024)	16	9.8	7.14 to 12.46	1.357	1.8418

Caffeine + music vs caffeine alone	Delleli et al. (2023)	16	0.66	−0.05 to 1.38	0.365	0.1331
Caffeine + music vs caffeine alone	Delleli et al. (2024)	16	1.42	0.63 to 2.20	0.401	0.1604
Caffeine + music vs music alone	Alsaeed et al. (2024)	16	2.18	1.28 to 3.07	0.457	0.2085
Caffeine + music vs music alone	Delleli et al. (2023)	16	2.77	1.77 to 3.77	0.51	0.2603
Caffeine + music vs music alone	Delleli et al. (2024)	16	2.35	1.42 to 3.28	0.474	0.2251
Comparison	Study	Paired n*	Paired SMD	95% CI	Paired SE†	Sampling variance
Caffeine + music vs placebo + no music	Qiu et al. (2025)	23	−0.25	−0.83 to 0.33	0.296	0.0876
Caffeine + music vs placebo + no music	Delleli et al. (2024)	16	0.49	−0.22 to 1.19	0.36	0.1294
Caffeine + music vs placebo + no music	Delleli et al. (2023)	16	4.8	3.37 to 6.23	0.73	0.5323
Caffeine + music vs caffeine alone	Delleli et al. (2024)	16	0	−0.69 to 0.69	0.352	0.1239
Caffeine + music vs caffeine alone	Delleli et al. (2023)	16	3.15	2.07 to 4.22	0.548	0.3008
Caffeine + music vs music alone	Qiu et al. (2025)	23	0.12	−0.46 to 0.69	0.293	0.0861
Caffeine + music vs music alone	Delleli et al. (2024)	16	−0.97	−1.71 to −0.24	0.375	0.1406
Caffeine + music vs music alone	Delleli et al. (2023)	16	2.54	1.58 to 3.50	0.49	0.2399
Comparison	Study	Paired n*	Paired SMD	95% CI	Paired SE†	Sampling variance
Caffeine + music vs placebo + no music	Qiu et al. (2025)	23	0.18	−0.40 to 0.76	0.296	0.0876
Caffeine + music vs placebo + no music	Delleli et al. (2024)	16	−2.93	−3.96 to −1.90	0.526	0.2762
Caffeine + music vs music alone	Qiu et al. (2025)	23	0.09	−0.48 to 0.67	0.293	0.0861
Caffeine + music vs music alone	Delleli et al. (2024)	16	−1.98	−2.85 to −1.12	0.441	0.1948
